# Maize WRKY Transcription Factor ZmWRKY106 Confers Drought and Heat Tolerance in Transgenic Plants

**DOI:** 10.3390/ijms19103046

**Published:** 2018-10-06

**Authors:** Chang-Tao Wang, Jing-Na Ru, Yong-Wei Liu, Meng Li, Dan Zhao, Jun-Feng Yang, Jin-Dong Fu, Zhao-Shi Xu

**Affiliations:** 1 Beijing Advanced Innovation Center for Food Nutrition and Human Health/Beijing Key Lab of Plant Resource Research and Development, Beijing Technology and Business University, Beijing 100048, China; wangct@th.btbu.edu.cn (C.-T.W.); limeng@th.btbu.edu.cn (M.L.); zhaodanustb@126.com (D.Z.); 2 Institute of Crop Science, Chinese Academy of Agricultural Sciences (CAAS)/National Key Facility for Crop Gene Resources and Genetic Improvement, Key Laboratory of Biology and Genetic Improvement of Triticeae Crops, Ministry of Agriculture, Beijing 100081, China; rujingna1993@163.com; 3 Institute of Genetics and Physiology, Hebei Academy of Agriculture and Forestry Sciences/Plant Genetic Engineering Center of Hebei Province, Shijiazhuang 050051, China; liuywmail@126.com; 4 Hebei Wangfeng Seed Industry Co., Ltd., Xingtai 054900, China; Yangjunfenghb@163.com

**Keywords:** WRKY, ZmWRKY106, drought tolerance, thermotolerance, maize

## Abstract

WRKY transcription factors constitute one of the largest transcription factor families in plants, and play crucial roles in plant growth and development, defense regulation and stress responses. However, knowledge about this family in maize is limited. In the present study, we identified a drought-induced WRKY gene, *ZmWRKY106*, based on the maize drought *de novo* transcriptome sequencing data. ZmWRKY106 was identified as part of the WRKYII group, and a phylogenetic tree analysis showed that ZmWRKY106 was closer to OsWRKY13. The subcellular localization of ZmWRKY106 was only observed in the nucleus. The promoter region of *ZmWRKY106* included the C-repeat/dehydration responsive element (DRE), low-temperature responsive element (LTR), MBS, and TCA-elements, which possibly participate in drought, cold, and salicylic acid (SA) stress responses. The expression of *ZmWRKY106* was induced significantly by drought, high temperature, and exogenous abscisic acid (ABA), but was weakly induced by salt. Overexpression of *ZmWRKY106* improved the tolerance to drought and heat in transgenic *Arabidopsis* by regulating stress-related genes through the ABA-signaling pathway, and the reactive oxygen species (ROS) content in transgenic lines was reduced by enhancing the activities of superoxide dismutase (SOD), peroxide dismutase (POD), and catalase (CAT) under drought stress. This suggested that *ZmWRKY106* was involved in multiple abiotic stress response pathways and acted as a positive factor under drought and heat stress.

## 1. Introduction

Changing environmental factors, such as abiotic stresses, influence plant growth and development [1]. Among them, drought and heat stresses seriously threaten crop productivity and quality. Plants must respond appropriately to changing environmental challenges to survive. Thus, it is important to explore the stress response mechanisms of plants and to enhance their tolerance to drought and heat to increase crop productivity without expanding cultivated land [2].

Environmental stresses initiate transcription factor (TF)-mediated expression of a variety of genes in plants, including bZIP, AP2/EREBP, MYB/MYC, NAC and WRKY [1,3,4,5,6]. WRKY TFs are identified by their conserved DNA-binding WRKY domains (WRKYGQK) in N-termini, and a zinc -finger motif (C-X4-5-C-X22-23-H-X1-H or C-X7-C-X23-H-X1-C) in C-termini [7,8]. It has been reported that WRKYs participated in defense responses by binding to the W-box located in the promoters of plant defense-related genes [9,10,11]. Another study found that 15 WRKY rice genes were induced by infection with the pathogen *Magnaporthe grisea* [12]. Statistically, 13 rice WRKY genes have regulated resistance against pathogens [13,14,15,16,17].

Recently, studies have revealed the involvement of the WRKY family in plant responses to abiotic stresses [18,19]. For example, *ABO3/WRKY63* took part in responses to abscisic acid (ABA) and drought stress in *Arabidopsis*. *AtWRKY57*-overexpressing *Arabidopsis* exhibited improved tolerance to drought by combining the promoter sequences of *NCED3* through the ABA pathway [20]. Overexpression of *OsWRKY30* enhanced resistance to drought stress in rice by the phosphorylation process of mitogen-activated protein kinases (MAPKs) [11]. *TaWRKY2* is a nuclear-located protein, and overexpression of *TaWRKY2* in *Arabidopsis* led to enhanced tolerance to drought and salt stresses by improving the expressions of *STZ* and *RD29B*; moreover, the exogenous expression of *TaWRKY19* in *Arabidopsis* not only conferred resistance to salt and drought, but also improved freezing tolerance [21]. In addition, *CmWRKY10*-overexpression in chrysanthemum revealed enhanced resistance to drought stress by regulating stress-related genes [19].

Maize (*Zea mays*) is a major food and economic crop. A few studies on the genome-wide analysis of WRKYs in maize have been reported in recent years. Wei et al. (2012) identified 136 WRKY proteins encoded by 119 *ZmWRKY* genes in maize, and Zhang et al. (2017) identified three additional new *ZmWRKY* genes and analyzed the gene expression profiles of *ZmWRKYs* using data from microarray, three RNA-seq studies, and the results of RT-PCR, which improved knowledge of WRKYs in maize [22,23]. In this paper, we performed drought-treated *de novo* transcriptome sequencing of maize (SRP144573) to investigate potential drought-tolerant WRKY genes in the maize genome. We identified a drought-responsive WRKY gene, *ZmWRKY106*, (Gene ID: GRMZM2G013391), which was named by Wei et al. (2012) and Zhang et al. (2017) [22,23]. The exogenous expression of *ZmWRKY106* in *Arabidopsis* led to enhanced tolerance of drought and heat.

## 2. Results

### 2.1. De Novo Transcriptome Sequencing Analysis

**Figure 1:**
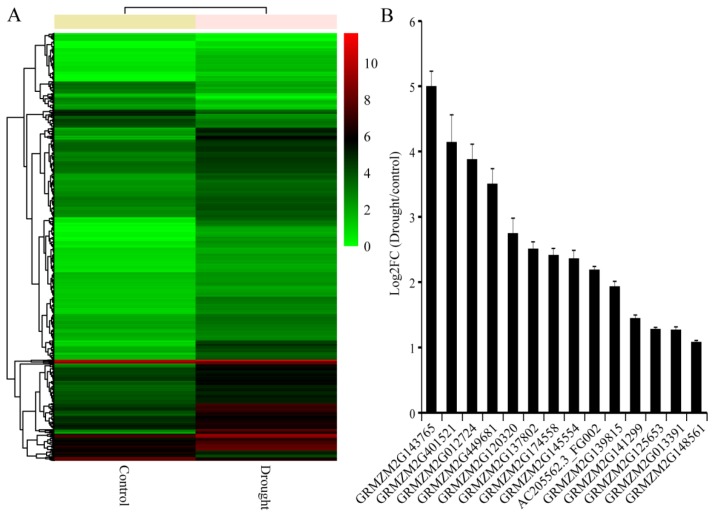
*De novo* transcriptome sequencing analysis of maize under drought stress. (**A**) Cluster analysis of the differentially expressed genes (DEGs) under drought treatment. (**B**) Transcription levels of the 14 differentially expressed *ZmWRKYs* under drought treatment. Error bar represent standard deviations (SD). The data represent means ± SD of three biological replications.

To find maize stress-responsive genes under drought stress, three-leaf seedlings were dehydrated on filter paper for 4 h, and then were collected for transcriptome sequencing analysis. The results showed that the transcription levels of many genes had changed after drought treatment (Figure 1A). Gene ontology (GO) analyses were used to classify the differentially expressed genes (DEGs) into functional groups. Almost 30 functionally enriched GO terms were identified for DEGs, and the results are shown in Supplementary Figure S1A. Among the predominantly enriched GO terms, signaling process was the most enriched term related to biological process. To further understand which pathways the stress-responsive genes may be involved in, the DEGs were analyzed against the Kyoto Encyclopedia of Genes and Genomes (KEGG) pathway database. The top 20 enriched pathways were identified, and the “plant hormone signal transduction” pathway enriched the most DEGs under drought treatment (Supplementary Figure S1B). DEGs including many transcription factors that play vital roles in plant growth, development, morphogenesis, and abiotic stress responses through regulating the expression of downstream genes [1,4,18]. Among these transcription factors, WRKYs play important roles in response to biotic and abiotic stresses [10,19]. We searched for *ZmWRKYs* among the DEGs, and found 14 *ZmWRKYs* induced by drought treatment (Figure 1B). We chose the gene GRMZM2G013391 named *ZmWRKY106* for further study. 

### 2.2. Phylogenetic Analysis of Maize ZmWRKY106

**Figure 2:**
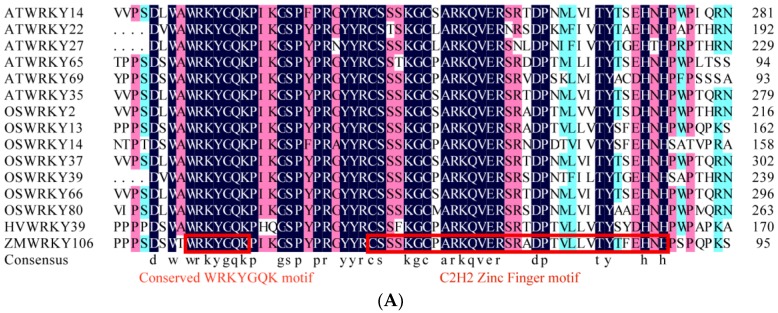

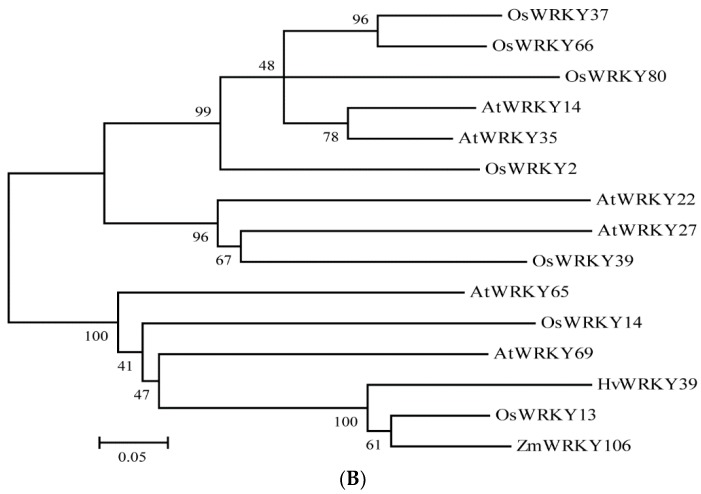
Multiple alignment and phylogenetic relationships of ZmWRKY106 with other orthologs in rice, *Arabidopsis*, and barley. The phylogenetic tree was produced using the aligned file with 1000 bootstraps in the MEGA 5.0 program. (**A**) Multiple alignment of ZmWRKY106 homologous proteins in rice, *Arabidopsis*, and barley. The different background colors represent the similar degree of amino acid sequences. (**B**) Phylogenetic relationship of ZmWRKY106 and other orthologs in different species. The first red box indicates the WRKYGQK motif, and the second indicates the conserved C2H2 zinc-finger motif.

After selecting from the drought-treated maize *de novo* transcriptome data, we got a putative WRKY gene *ZmWRKY106* encoding 277 amino acids. The BLASTp online tool was used to search for the homologous amino acid sequences of *ZmWRKY106* in rice and *Arabidopsis*. The amino acid sequence alignment and phylogeny analysis of ZmWRKY106 orthologs are shown in Figure 2. ZmWRKY106 shared a mean identity of 28.47% with its rice, *Arabidopsis*, and barley orthologs and had a conserved signature WRKYGQK at the N-terminus followed by a C2H2 zinc-finger motif (C-X5-C-X23-H-X1-H), which characterized group II (Figure 2A). The sequences outside the conserved domain/motif were very different. The results of phylogenesis showed that ZmWRKY106 was closer to OsWRKY13, with a 61% bootstrap rate, followed by HvWRKY39, with a frequency of 100% (Figure 2B). However, the identity of ZmWRKY106 with other orthologs was lower than that with OsWRKY13, which indicated that ZmWRKY106 may have an extensive difference from other members.

### 2.3. ZmWRKY106 Was Localized in the Nucleus

**Figure 3:**
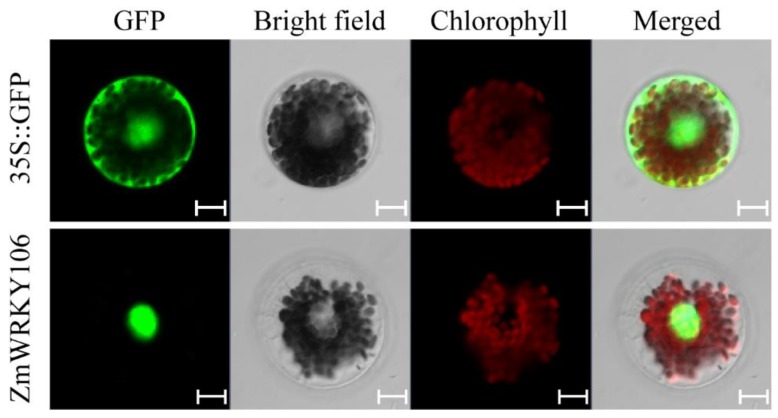
Subcellular localization of *ZmWRKY106*. The p16318hGFP and p16318hGFP-ZmWRKY106 constructs were transiently expressed in maize protoplasts. The green indicates green fluorescent, and the red indicates chloroplast autofluorescence. Results were observed after transformation for 18 h with confocal microscopy. Scale bars = 10 μm.

The transient expression vector p16318h-ZmWRKY106 was transformed to maize mesophyll protoplasts by the PEG-mediated method to determine the cell localization. After incubation in darkness for 18 h, the fluorescence signals were monitored by a confocal laser scanning microscope. As shown in Figure 3, relative to the control distributed throughout the cell, the p16318h-ZmWRKY106 fusion protein was specifically detected in the nucleus.

### 2.4. ZmWRKY106 Promoter Domain Contained Various Stress-Related Cis-Elements

To further understand the regulation mechanism of *ZmWRKY106*, we isolated the promoter region upstream of the *ZmWRKY106* ATG start codon. Types of *cis*-elements correlated to stress were present in the promoter region, including the C-repeat/DRE element referred to cold and dehydration response, low-temperature responsive element LTR and the drought-induced element MBS. In addition, there was another TCA-element that participated in salicylic acid (SA) response in the promoter region of *ZmWRKY106* (Table 1). This analysis suggested that *ZmWRKY106* may function in abiotic stress response.

### 2.5. ZmWRKY106 Was Involved in Abiotic Stress Responses

**Figure 4:**
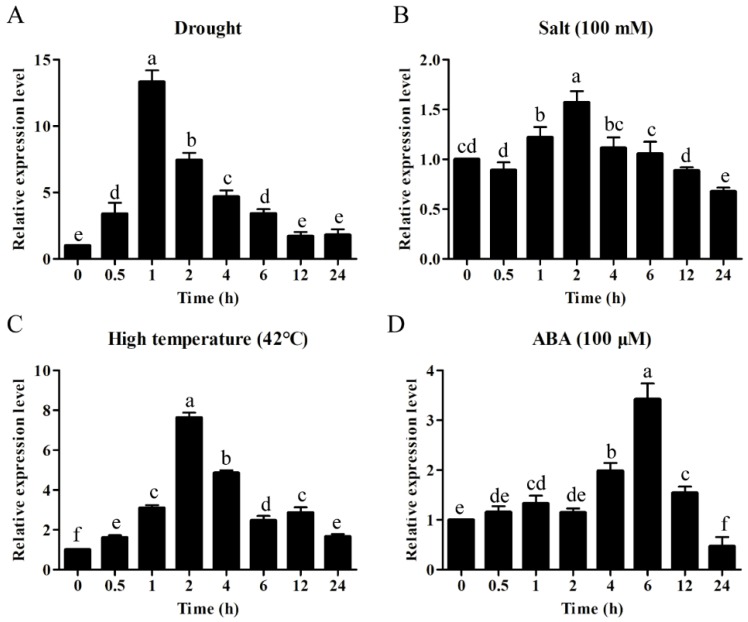
Expression patterns of *ZmWRKY106* under (**A**) drought, (**B**) high-salt, (**C**) high-temperature, and (**D**) exogenous abscisic acid (ABA) stresses. The ordinates are the relative expression level (fold) of *ZmWRKY106* compared to the non-stressed control. The horizontal ordinate is treatment time for 0, 0.5, 1, 2, 4, 6, 12 and 24 h. All experiments were repeated three times. Error bars represent standard deviations (SDs). All the data represent the means ± SDs of three independent biological replicates. The different letters in the bar graphs indicate significant differences at *p* < 0.05.

To explore the possible signal pathways which *ZmWRKY106* may be involved in, we performed qRT-PCR to investigate the expression patterns of *ZmWRKY106* in maize treated with drought, high-salt, high-temperature, and ABA treatments. *ZmWRKY106* was remarkably induced by drought, high temperature and ABA, but was weakly induced by salt (Figure 4). For dehydration treatment, the transcript of *ZmWRKY106* was rapidly up-regulated more than 10-fold after 1 h of dehydration stress (Figure 4A). *ZmWRKY106* was slightly induced by salt at a maximum level of about 1.5-fold (Figure 4B). High temperature also significantly affected the expression of *ZmWRKY106*. Under high-temperature stress, the transcription level of *ZmWRKY106* increased gradually, peaked at 7.6-fold after 2 h of stress, and then rapidly declined to a constitutive level. With exogenous ABA treatment, the transcription level of *ZmWRKY106* was increased more than three-fold at 6 h after treatment. 

### 2.6. ZmWRKY106 Enhanced Drought Tolerance in Transgenic Arabidopsis

**Figure 5:**
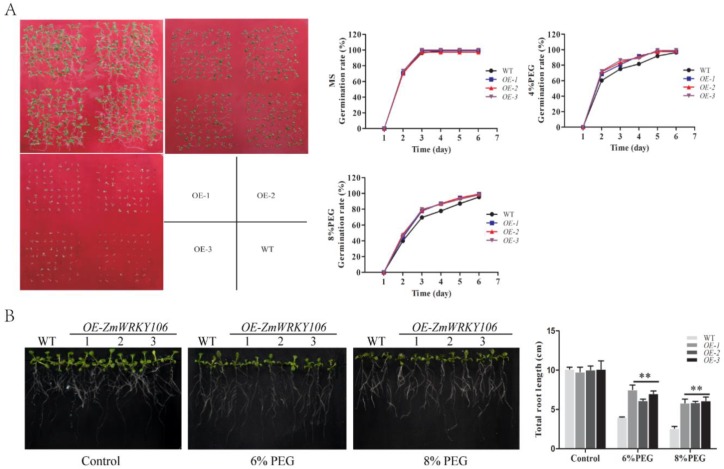
Phenotypes of *ZmWRKY106* transgenic *Arabidopsis* under drought treatment. (**A**) Seed germinations of wild-type (WT) and *ZmWRKY106*-overexpressing lines. (**B**) Root lengths of WT and *ZmWRKY106* transgenic plants. Five-day-old seedlings were transferred to MS medium supplemented with or without PEG6000 for seven days, and then root lengths were measured. All the data represent the means ± SDs of three independent biological replicates and asterisks (**) represent the significant differences at *p* < 0.01 (Student’s *t*-test).

To investigate the function of *ZmWRKY106*, the pBI121-*ZmWRKY106* recombinant was transformed into wild-type (WT) *Arabidopsis* (Columbia-0). T_3_ generation transgenic lines with relatively high expressions were selected by qRT-PCR for further analysis. The expression levels of transgenic lines are exhibited in Supplementary Figure S2. On MS medium, no significant differences in seed germination rates were observed between transgenic and WT plants. In the presence of 4% PEG6000, the germination rate of transgenic seeds was nearly 9% higher than WT after four days. Moreover, the germination was suppressed under 8% PEG6000, but transgenic seeds showed a higher germination rate than WT seeds (Figure 5A). For root growth assays, as shown in Figure 5B, *ZmWRKY106* transgenic lines had similar phenotypes to WT on MS medium. When supplemented with PEG6000, the growth of all transgenic and WT plants was repressed; however, transgenic plants showed clear differences compared to WT ones, with significantly longer total root lengths than those of WT under both PEG treatments. These results showed that *ZmWRKY106* transgenic lines had a stronger capacity to resist drought.

### 2.7. ZmWRKY106 Enhanced Heat Tolerance in Transgenic Arabidopsis

**Figure 6:**
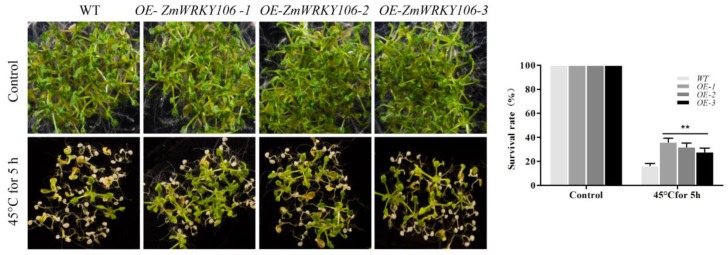
Survival rates of WT and *ZmWRKY106* transgenic lines under heat stress. Five-day-old seedlings were placed at 45 °C for 5 h and then resumed growth at 22 °C. The data represent the means ± SDs of three independent biological replicates. Asterisks (**) represent the significant differences (*p* < 0.01) compared with the control (Student’s *t*-test).

Under high temperature, the expression of *ZmWRKY106* was up-regulated. Following this result, we observed the phenotypes among WT and transgenic lines under 45 °C (Figure 6). The survival rates of transgenic and WT plants were 100% under normal conditions, while higher a survival rate was exhibited in OE lines than WT after heat treatment for 5 h. *ZmWRKY106*-overexpressing lines had a survival rate of more than 30%, compared to less than 20% for WT plants after heat treatment. This suggested that *ZmWRKY106* may improve thermotolerance of transgenic plants. 

### 2.8. ZmWRKY106 Regulated the Expression of Stress-Related Genes

**Figure 7:**
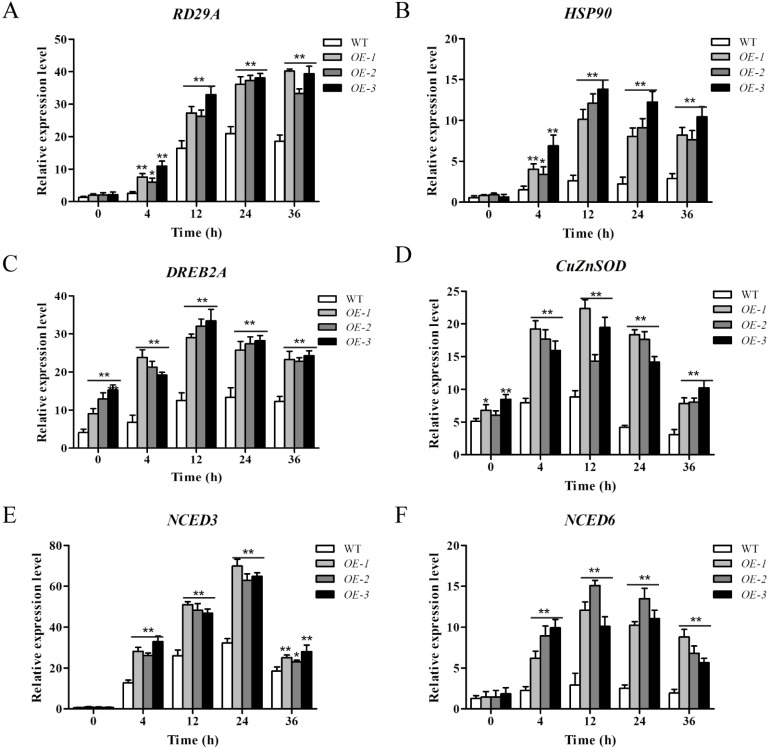
The relative expression of stress-related genes. (**A**) *RD29A*, (**B**) *HSP90*, (**C**) *DREB2A*, (**D**) *CuZnSOD*, (**E**) *NCED3*, and (**F**) *NCED6* were examined under control and drought conditions for various time points (4, 12, 24 and 36 h). Values are means ± SDs of three replicates, and asterisks (* or **) represent the significant differences at *p* < 0.05 or *p* < 0.01, respectively (Student’s *t*-test).

To understand the molecular mechanisms of *ZmWRKY106* in stress responses, expression of stress-responsive genes, including *RD29A*, *HSP90*, *DREB2A*, *CuZnSOD*, *NCED3*, and *NCED6*, was examined using qRT-PCR under normal and drought conditions. The results showed that the expression levels of *HSP90* and *NCED3* were low in both WT and OE lines under normal conditions, while the expression levels in OE lines remained higher than WT plants after treatment (Figure 7B,E). Meanwhile, *CuZnSOD* and *NCED6* in OE lines were up-regulated after 4 h of stress treatment, and sharply increased to the maximum (Figure 7D,F). The expression levels of *RD29A* and *DREB2A* in OE lines were remarkably higher following all treatments (Figure 7A,C). Because the expressions of ABA and stress-related genes were altered in transgenic lines, we conjectured that *ZmWRKY106* may play a role in the abiotic stress response by regulating stress-related genes through the ABA-signaling pathway.

### 2.9. Overexpression of ZmWRKY106 Reduced Reactive Oxygen Species (ROS) Content and Enhanced the Activities of Superoxide Dismutase (SOD), Peroxide Dismutase (POD), and Catalase (CAT) under Drought Treatment

**Figure 8:**
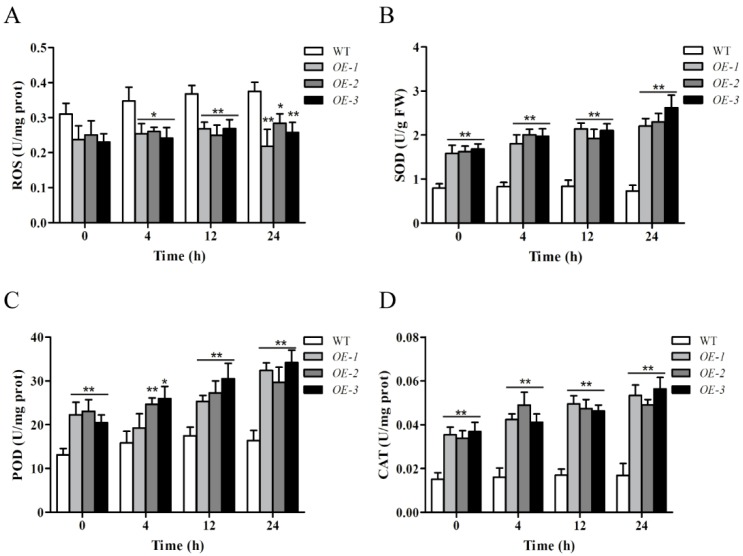
(**A**) The reactive oxygen species (ROS) content and the activities of (**B**) superoxide dismutase (SOD), (**C**) peroxide dismutase (POD), and (**D**) catalase (CAT) under different conditions at different time points (0, 4, 12, and 24 h). Values are means ± SDs of three replicates, and asterisks (* or **) represent the significant differences at *p* < 0.05 or *p* < 0.01, respectively (Student’s *t*-test).

The ROS content and the enzyme activities were assessed in transgenic lines and WT plants at 0, 4, 12 and 24 h after drought treatment (Figure 8). As shown in Figure 8A, the ROS accumulation in transgenic lines was less than that in WT plants at all times, while the ROS content was increased in WT plants and remained at a higher level during the whole experiment. The activities of SOD, POD and CAT were increased in OE lines compared to WT lines (Figure 8B–D). The activity of SOD was almost unchanged in WT before and after drought treatment, whereas in OE lines the activity of SOD was greater, and reached a maximum at 24 h after drought treatment (Figure 8B). Increases of POD activity were observed in both WT and transgenic lines, but the increases in WT were smaller, and there was consistently higher POD activity in transgenic lines than in WT lines (Figure 8C). In the case of CAT, the CAT activity of WT lines remained consistent at 0.016 U and had almost no significant change during the stress treatment; however, the CAT concentration in the OE lines remained significantly higher compared to that in WT lines under drought stress (Figure 8D). In a word, overexpression of *ZmWRKY106* reduced ROS content by enhancing the activities of SOD, POD and CAT to resist drought stress. 

## 3. Discussion

Biotic and abiotic stresses seriously affect plant growth and development. Under adverse environments, transcriptome changes are the earliest responses, and transcriptional regulation plays a crucial role in plant defense responses [15]. Thus far, many TFs have been identified as participating in plant defense responses, including MYB, bZIP, and WRKY proteins. There are many more biotic stress-related genes in WRKYs than in other TFs, and an increasing number of studies have revealed that WRKY TFs play positive or negative roles in plants’ disease prevention [24]. For example, *AtWRKY46*, coordinated with *AtWRKY70* and *AtWRKY53*, positively regulated basal resistance to *Pseudomonas syringae* [25]; *OsWRKY6* played a positive role in plant defense response by activating the expression of defense-related genes [26]; *GhWRKY44* was induced by pathogen injection, and overexpression of *GhWRKY44* led to enhanced resistance against bacterial and fungal pathogens [27]. These results all suggest that the WRKY family plays an important role in responding to biotic stresses [28].

However, knowledge about the role of WRKYs in abiotic stresses is limited [29,30]. Maize is a major food and economic crop and plays an important role in basic and applied biological research. So far, known research about WRKYs has been mostly related to defense response in dicotyledon plants such as *Arabidopsis*, tomato, and tobacco, but little information about the role of maize WRKYs has been reported [31,32,33]. It is rather crucial to elucidate the functional maize WRKY protein in abiotic stress response. In maize, Wei et al. [22] have identified 136 WRKY proteins encoded by 119 WRKY genes, numbered them, and performed a phylogenetic tree analysis of the maize WRKYs with orthologs in *Arabidopsis*, rice, and barley, which improved knowledge of WRKYs in maize. In addition, Zhang et al. [23] identified three new additional *ZmWRKY* genes, analyzed the gene expression profiles of *ZmWRKYs* using data from various studies, and found that ten genes, including *ZmWRKY9*, *ZmWRKY25*, *ZmWRKY47*, *ZmWRKY97*, *ZmWRKY80*, *ZmWRKY39*, *ZmWRKY106*, *ZmWRKY53*, *ZmWRKY36* and *ZmWRKY113*, were responsive under drought treatment in at least in three studies, which provided the basis for cloning functional *ZmWRKY* genes. In this study, we revealed the function of *ZmWRKY106* in abiotic stress responses. Our study showed that ZmWRKY106 belongs to group II, shares a mean identity with its rice, *Arabidopsis* and barley orthologs, and is closer to *OsWRKY13* (Figure 2).

Increasing evidence has indicated that WRKYs play an important role in abiotic stress response, for example, *GmWRKY21* improved freezing tolerance in transgenic *Arabidopsis*, and *GmWRKY54* played a positive role in response to salt and drought stresses, whereas *GmWRKY13* markedly increased sensitivity to salt and mannitol [34]. Overexpression of *AtWRKY25* and *AtWRKY33* in *Arabidopsis* led to enhanced resistance to salt and hypersensitivity to ABA [35]. In rice, *OsWRKY11* enhanced heat and drought tolerance [29]. In barley, *Hv-WRKY38* played key roles in the response to cold and drought stresses, and enhanced drought tolerance in turf and forage grass [36,37]. In this study, expression profiles analysis revealed that *ZmWRKY106* was induced significantly by drought, high temperature and ABA (Figure 4), possibly related to various stress-related cis-elements in its promoter region (Table 1). Under drought treatment, the transgenic seeds of *ZmWRKY106* germinated faster than WT seeds, and roots of OE lines were remarkably longer than those of WT lines (Figure 5). Meanwhile, overexpression of *ZmWRKY106* reduced ROS content and enhanced the activities of SOD, POD and CAT under drought treatment (Figure 8). Furthermore, the survival rates of OE lines were higher than those of WT lines (Figure 6). These results all showed that *ZmWRKY106* exhibited drought tolerance and thermotolerance.

ABA is a major phytohormone referred to plant response under drought stress, and there exist ABA-dependent and ABA-independent pathways in drought stress response. In our study, the expression levels of six stress-related genes were assessed under normal and drought conditions (Figure 7). *DREB2A* is a well-known marker gene in ABA-independent stress responses [38]. ABRE and DRE/CRT motifs were found in the promoters of many stress-inducible genes, such as *RD29A*, which contained several DREs and one ABRE in the promoter domain, and was strongly induced by cold, drought and salt stresses [39,40,41]. *HSP90* played a major role in stress signal transduction, and overexpression of HSP90 affected the phenotype of transgenic plants [42,43,44,45]. In our study, the expressions of *RD29A*, *HSP90*, and *DREB2A* genes were all up-regulated in *ZmWRKY106* transgenic lines (Figure 7A–C), suggesting that *ZmWRKY106* may play a positive role in drought and heat response. *CmWRKY10* acted as a positive factor in response to drought stress by regulating the expression of *DREB1A*, *DREB2A*, *CuZnSOD*, *NCED3A*, and *NCED3B*, which proved that *CmWRKY10* enhanced the drought tolerance through the ABA-dependent pathway [19]. These genes could play key roles in the physiological process of abiotic stress response [46,47]. We found that the expressions of ABA-related genes were higher in transgenic lines, which indicated that overexpression of *ZmWRKY106* led to enhanced tolerance of drought stress through the ABA-dependent pathway (Figure 7D–F). These results all indicated that *ZmWRKY106* may play a role in the abiotic stress response by regulating stress-related genes through the ABA-signaling pathway (Figure 7). Nevertheless, the role and regulation mechanisms of *ZmWRKY106* in maize still need further research.

## 4. Materials and Methods

### 4.1. De Novo Transcriptome Sequencing

Three-leaf stage untreated maize seedlings and seedlings dehydrated on filter paper for 4 h were collected for RNA-seq analysis. The detailed process of RNA-seq was undertaken as previously described [18]. The transcriptome data are available in the National Center for Biotechnology Information (NCBI) under accession number SRP144573.

### 4.2. Plant Materials and Stress Treatments

The seeds of maize (X178) used in this study were provided by Zhuan-Fang Hao (Institute of Crop Science, Chinese Academy of Agricultural Sciences, Beijing, China). The maize seeds were sown as previously described [48]. Three-leaf stage maize seedlings were exposed to drought, salt, high-temperature and ABA treatments. For dehydration treatment, seedlings were quickly cleaned and then transferred on to filter paper to rapidly dry in air as previously described [49]. Seedlings were placed in 42 °C chambers for high-temperature treatment. For salt and ABA treatments, seedlings were exposed to water solutions supplemented with 100 mM NaCl, and 100 μM ABA, respectively. The samples were collected at 0, 0.5, 1, 2, 4, 6, 12 and 24 h after treatment. Harvested seedlings were dropped immediately into liquid nitrogen and stored at −80 °C for RNA extraction. 

### 4.3. RNA Extraction and Quantitative Real-Time PCR (qRT-PCR)

Total RNAs were extracted from maize tissue using an RNAprep plant kit (Tiangen, Beijing, China), and cDNA was synthesized as previously described [18]. The qRT-PCR was performed with SuperReal PreMix Plus (Tiangen, Beijing, China) by an ABI Prism 7500 system (Applied Biosystems, Foster City, CA, USA). The specific primers of *ZmWRKY106* are listed in Supplementary Table S1. Each PCR was repeated three times and data were analyzed, as previously described [50].

### 4.4. Gene Isolation and Sequence Analysis

The full length of the *ZmWRKY106* gene was amplified by PCR with specific primers from maize cDNA. The primers of *ZmWRKY106*-F and *ZmWRKY106*-R are listed in Supplementary Table S1. The PCR products were cloned into pLB vector (Tiangen, China) and sequenced. The homologs of *ZmWRKY106* in different species were searched for in the NCBI database. Sequence alignments of *ZmWRKY106* orthologs were performed by ClustalX software. The phylogenetic tree was constructed using the neighbor-joining method by the MEGA 5.0 program with bootstrap analysis of 1000 replicates [51].

### 4.5. Subcellular Localization

The coding region of *ZmWRKY106* was fused to the subcellular localization vector p16318h with green fluorescent protein (GFP) tags containing the CaMV35S promoter. The specific primers are listed in Supplementary Table S1. For transient expression assays, the p16318h-*ZmWRKY106* reconstruction plasmid was transformed to maize mesophyll protoplasts by the PEG-mediated method, while the p16318hGFP vector was transformed as control [52]. The fluorescence signals were observed by a confocal laser scanning microscope (LSM700; CarlZeiss, Oberkochen, Germany) after incubation in darkness at 22 °C for 18 h.

### 4.6. Cis-Acting Elements in Promoter

The 2.0 kb promoter region upstream of *ZmWRKY106* was obtained from maize genomic DNA on the EnsemblPlants website (available online: http://plants.ensembl.org/index.html). Putative cis-acting elements in the promoter region were analyzed using the PLACE database [53].

### 4.7. Generation of Transgenic *Arabidopsis* and Its Phenotype under Stress Treatment

Plant expression vector pBI121-*ZmWRKY106* was constructed as previously described [54], and was transformed to wild-type (WT) *Arabidopsis* using the *Agrobacterium*-mediated floral dip method. Columbia-0 (WT) was used for exogenous expression of *ZmWRKY106*. The transformed seeds were selected on MS medium containing 50 mM Kanamycin at 22 °C with a photoperiod of 16 h light/8 h dark (60% humidity) to obtain the positive plants. Three T_3_ generation overexpression lines (*OE-ZmWRKY106-1*, *OE-ZmWRKY106-2*, *OE-ZmWRKY106-3*) with higher expression levels of *ZmWRKY106* were selected by qRT-PCR for further analysis. *Arabidopsis* seeds were grown as described previously [50]. Four-week-old seedlings of transgenic and WT *Arabidopsis* were collected at 0, 4, 12, 24 and 36 h after drought treatment (dried on filter paper) to examine the expression of stress-related genes by qRT-PCR. The specific primers of stress-related genes are listed in Supplementary Table S1. Three biological replicates were performed for qRT-PCR. 

For the germination assay, WT and transgenic *Arabidopsis* seeds were placed on MS medium and MS medium supplemented with 4% (*w*/*v*) or 8% PEG6000. When the radicle had emerged from the seed coat, we considered the seed germinated. Seed germination was followed for five days, and the germination rate was analyzed. For the root growth assay, five-day-old seedlings were transferred to MS medium with or without 6% and 8% PEG6000 for seven days, and then root lengths were measured. Each treatment contained three independent replicates.

For high-temperature stress assay, five-day-old seedlings were placed at 45 °C for 5 h and then resumed growth at 22 °C as described previously [18]. After growing under normal conditions for seven days, we took photos and analyzed the survival rate. Each treatment contained three independent replicates. Values are means ±SD and statistically significant differences were based on the Student’s test.

### 4.8. Measurements of Reactive Oxygen Species (ROS) Content and Enzyme Activity

To better understand the function of *ZmWRKY106* under drought treatment, we assessed the activities of superoxide dismutase (SOD), peroxide dismutase (POD), catalase (CAT) and the ROS content in WT and transgenic lines at 0, 4, 12 and 24 h after drought stress. The ROS content and the activities of SOD, POD, and CAT were measured as previously described [19]. To obtain reproducible results, each experiment was repeated three times. Values are means ±SD and statistically significant differences were based on the Student’s test.

## 5. Conclusions

We identified a drought-induced WRKYII gene *ZmWRKY106* based on maize drought *de novo* transcriptome sequencing data (SRP144573). *ZmWRKY106* was only observed in the nucleus. The expression of *ZmWRKY106* was induced significantly by drought, high-temperature, and exogenous abscisic acid (ABA) treatments, but was induced weakly by salt. Further research revealed that overexpression of *ZmWRKY106* could improve tolerance to drought and heat in transgenic *Arabidopsis* by regulating stress-related genes through the ABA-signaling pathway, and could reduce the ROS content in transgenic lines by enhancing the activities of SOD, POD and CAT under drought stress. These results may provide a basis for understanding the functions of *ZmWRKY106* in abiotic stress response in maize.

## Figures and Tables

**Table 1:** Putative *cis*-elements in the *ZmWRKY106* promoter.

Elements	Sequence	Function
C-repeat/DRE	TGGCCGAC	involved in cold- and dehydration-responsiveness
LTR	CCGAAA	involved in low-temperature responsiveness
MBS	TAACTG	MYB binding site involved in drought-inducibility
TCA-element	TCAGAAGAGG	involved in SA responsiveness

DRE—dehydration responsive element; LTR—low-temperature responsive; SA—salicylic acid.

## Supplementary Materials

Supplementary materials can be found at http://www.mdpi.com/1422-0067/19/10/3046/s1.

## Abbreviations

ABA	abscisic acid
ABRE	ABA-responsive element
DRE	dehydration responsive element
GFP	green fluorescent protein
LTR	low-temperature responsive
MAPKs	mitogen-activated protein kinases
qRT-PCR	quantitative real-time PCR
RT-PCR	reverse transcription PCR
SA	salicylic acid
TF	transcription factor
WT	wild type
