# Supplementary material for: Maize WRKY Transcription Factor ZmWRKY106 Confers Drought and Heat Tolerance in Transgenic Plants
Source: Int J Mol Sci. 2018 Oct 6;19(10):3046. doi: 10.3390/ijms19103046 (PMC6213049; doi:10.3390/ijms19103046)
Supplement: Supplementary file 1 [file ijms-19-03046-s001.zip › Supplementary metarials/Supplemntary Table S1.docx]

| **Table S1. Primers used in the paper.** | | | |
| --- | --- | --- | --- |
| **Constructions** | | **Primer sequences** |  |
| **Primers for qRT-PCR analysis** | | |  |
| *Zm-Actin*-F | | GCATCCATGAGACCACCTACAAC |  |
| *Zm-Actin*-R | | GATGGACCCTCCTATCCAGACAC |  |
| *RT-ZmWRKY106*-F | | GCTCGTCACCTACACCTTCG |  |
| *RT-ZmWRKY106*-R | | AGCTTTCGTCCTCCTCTGC |  |
| *At-Actin*-F | | GAAATCACAGCACTTGCACC |  |
| *At-Actin*-R | | AAGCCTTTGATCTTGAGAGC |  |
| *AtRD29A*-F | | GTCTGCCGTGACGACGAAGTTAC |  |
| *AtRD29A*-R | | TCCTTCTTCTCTTCTTCTCCTCCAA |  |
| *AtHSP90*-F | | TGGCAGACTGATTGGGAGTG |  |
| *AtHSP90*-R | | CTGTCTCAAAAACATCGGCACA |  |
| *AtDREB2A*-F | | ATGGGAAACCTGGGGAGAAAG |  |
| *AtDREB2A*-R | | AAACCGTGTGGTGGCCTTC |  |
| *AtCuZnSOD*-F | | GCTGATCCACCAAGGAAAGGTT |  |
| *AtCuZnSOD*-R | | GTGGCTGTGTAGCACTAGGT |  |
| *AtNCED3*-F | | ACAGCCTCGTCCCTAAGTCT |  |
| *AtNCED3*-R | | GCCCTCCCTCCTAAAGTGAC |  |
| *AtNCED6*-F | | GGAGGACAGCAGGAGCCTAA |  |
| *AtNCED6*-R | | CTGTGAAACCCATGTCCCTGAA |  |
| **Primers for gene amplification** | | |  |
| *ZmWRKY106*-F | | CCACCATCAGGGCGAAAAT |  |
| *ZmWRKY106*-R | | CGCAAACGGGACAAACGA |  |
| **Primers for subcellular localization** | | |  |
| p16318-*ZmWRKY106*-F |  | TATCTCTAGAGGATCCATGTCGTCGGGC |  |
| p16318-*ZmWRKY106*-R |  | TGCTCACCATGGATCCGGAGCACGGCGC |  |
| **Primers for transgenic *Arabidopsis*** | | |  |
| pBI121-*ZmWRKY106*-F | | CTCTAGAGGATCCCCGGGATGTCGTCGGGC |  |
| pBI121-*ZmWRKY106*-R | | ACTAGTGGATCCCCCGGGTCAGGAGCACGG |  |
