# Supplementary figures and images for: Maize WRKY Transcription Factor ZmWRKY106 Confers Drought and Heat Tolerance in Transgenic Plants
Source: Int J Mol Sci. 2018 Oct 6;19(10):3046. doi: 10.3390/ijms19103046 (PMC6213049; doi:10.3390/ijms19103046)

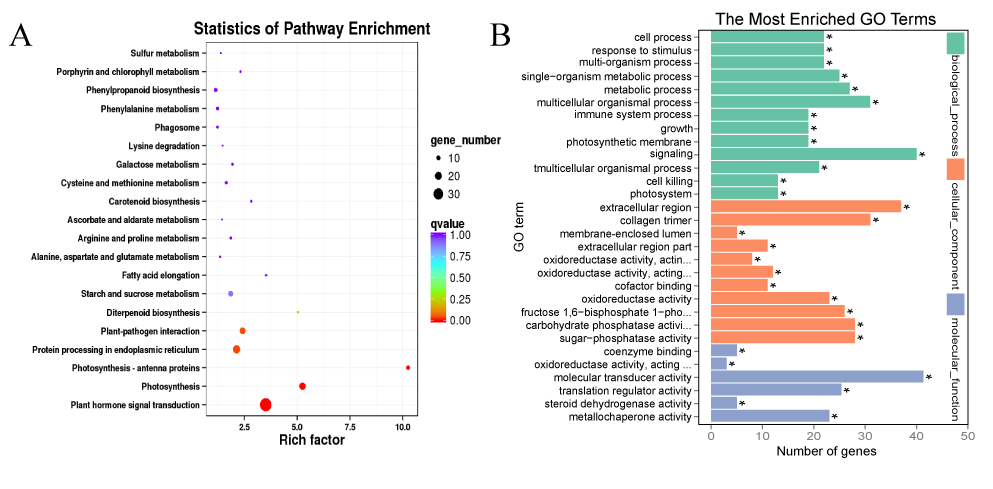

Supplement: Supplementary file 1 [file ijms-19-03046-s001.zip › Supplementary metarials/Supplementary Figure S1.tif]

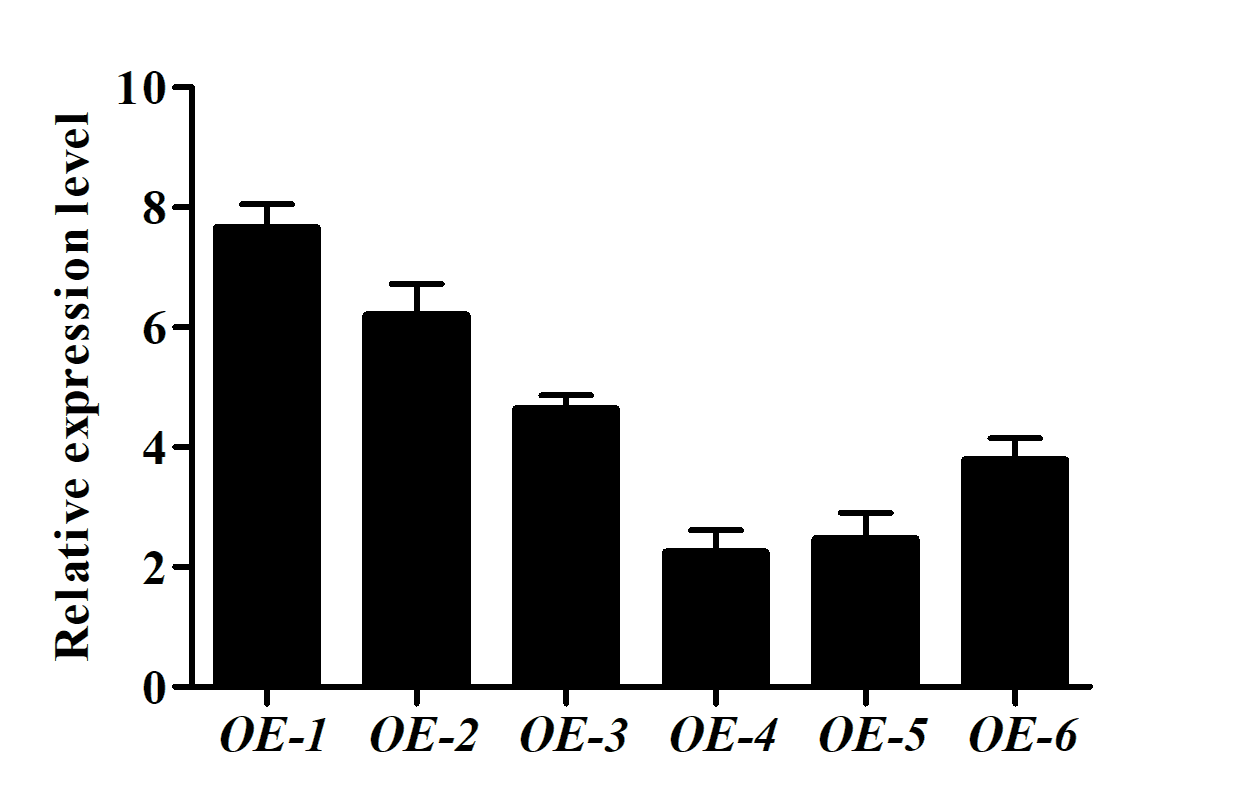

Supplement: Supplementary file 1 [file ijms-19-03046-s001.zip › Supplementary metarials/Supplementary Figure S2.tif]
